# Migrating glioma cells express stem cell markers and give rise to new tumors upon xenografting

**DOI:** 10.1007/s11060-016-2221-y

**Published:** 2016-08-10

**Authors:** Sune Munthe, Mia D. Sørensen, Mads Thomassen, Mark Burton, Torben A. Kruse, Justin D. Lathia, Frantz Rom Poulsen, Bjarne Winther Kristensen

**Affiliations:** 1 Department of Neurosurgery, Odense University Hospital, Sdr. Boulevard 29, 5000 Odense, Denmark; 2 Department of Pathology, Odense University Hospital, Winsloew parken 15, 5000 Odense, Denmark; 3 Institute of Clinical Research, University of Southern Denmark, Odense, Denmark; 4 Department of Clinical Genetics, Odense University Hospital, Odense, Denmark; 5 Department of Cellular and Molecular Medicine, Lerner Research Institute, Cleveland Clinic, Cleveland, OH 44195 USA; 6 Department of Cellular and Molecular Medicine, Lerner College of Medicine of Case Western Reserve University, Cleveland, OH 44195 USA

**Keywords:** Glioblastoma, Migration, Invasion, Cancer stem cell

## Abstract

Glioblastoma (GBM) is the most frequent and malignant brain tumor with an overall survival of only 14.6 months. Although these tumors are treated with surgery, radiation and chemotherapy, recurrence is inevitable. A critical population of tumor cells in terms of therapy, the so-called cancer stem cells (CSCs), has been identified in gliomas and many other cancers. These tumor cells have a stem cell-like phenotype and are suggested to be responsible for tumor growth, chemo- and radio-resistance as well as recurrence. However, functional evidence for migrating glioma cells having a stem cell-like phenotype is currently lacking. In the present study, the aim was to characterize the phenotype of migrating tumor cells using a novel migration assay based on serum-free stem cell medium and patient-derived spheroid cultures. The results showed pronounced migration of five different GBM spheroid cultures, but not of the commercial cell line U87MG. An in vitro limiting dilution assay showed preserved but reduced spheroid formation capacity of migrating cells. Orthotopic xenografting in mice showed preserved but reduced tumorigenic capacity. Profiling of mRNAs revealed no significant deregulation of 16 predefined CSC-related genes and the HOX-gene list in migrating cells compared to spheroids. Determination of GBM molecular subtypes revealed that subtypes of spheroids and migrating cells were identical. In conclusion, migrating tumor cells preserve expression of stem cell markers and functional CSC characteristics. Since CSCs are reported to be highly resistant to therapy, these results emphasize that the CSC phenotype should be taken into consideration in future treatment of GBMs.

## Introduction

Glioblastoma (GBM) is the most frequent primary malignant central nervous system tumor. Despite multi-modal therapy including surgery, irradiation and chemotherapy, the median survival is only 14.6 months [1, 2]. GBM tumor cells migrate into the normal brain parenchyma along vessels and white matter fiber-tracts [3]. The highly migratory capability is thought to be a major reason for the short overall survival of GBM patients, since it results in tumor cells being left behind after surgery. However, the phenotype of migrating tumors cells is poorly described thereby preventing development of efficient therapies. 

A population of tumor cells with stem cell characteristics has been described in GBM and many other cancers [4, 5]. These self-renewing cancer stem cells (CSCs) have been suggested to be responsible for tumor growth as well as chemo- and radio-resistance and recurrence [6, 7]. According to expression of stem cell markers, we have recently shown that migrating tumor cells in glioma biopsy material display a stem cell phenotype [8]. However, the functional evidence for tumor initiating capabilities of these migrating cells is currently lacking.

Different in vitro migration assays have previously been used to study migrating glioma cells, but these migration assays are based on fetal calf serum as chemoattractant [9–12]. A limitation with this approach is that tumor cells undergo changes in phenotype including differentiation into tumor cells expressing astrocytic, oligodendroglial and neural markers when exposed to fetal calf serum [13, 14]. Using a novel in vitro migration assay based on patient-derived GBM spheroid cultures grown in a chemically defined serum-free medium, we hypothesized that migrating tumor cells had a stem cell phenotype. The CSC phenotype of isolated migrating cells were investigated using limiting dilution spheroid formation assay as well as using orthotopic xenografting at decreasing tumor cell concentrations to investigate tumor-initiating capabilities [14–18]. Using mRNA profiling of both migrating and sphere cells, two predefined stem cell or CSC-related gene-lists were evaluated. The recently established molecular GBM subtypes originally described by Verhaak et al. were also addressed [19]. A potential change in subtype induced by migration has not previously been addressed. We hypothesized that this might occur and investigated this by the mRNA profiling data.

## Material and methods

### Cell culture

GBM cells including the U87MG cell line were cultured as free-floating spheroids in serum-free neural stem cell medium [16] at 36 °C in a humidified incubator with 5 % CO_2_. The spheroid cultures were established in our laboratory as previously described from patient-derived tissue [18]. All spheroid cultures had a karyotype typical of GBMs and the ability to form new spheroids at clonal density. Moreover, they differentiated into cells expressing neuronal, astrocytic and oligodendrocyte markers upon culturing in serum-containing medium. They formed highly invasive tumors upon orthotopic xenografting. All GBM spheroid cultures (T78, T87 and T111) except the T86 and T113 culture [16, 18] had a hypermethylated O6-methylguanine-DNA methyltransferase (MGMT) promoter region and were derived from GBMs without mutated isocitrate dehydrogenase 1 (mIDH1). In the present study, the GBM spheroid cultures were used in the current study at the following passage (P) numbers: T78, P14; T86, P15; T87, P13; T111 P16; T113, P14. Use of human tissue in the present study was approved by the Regional Scientific Ethical Committee (approval number S-VF-20040102).

### Migration assay

**Fig. 1:**
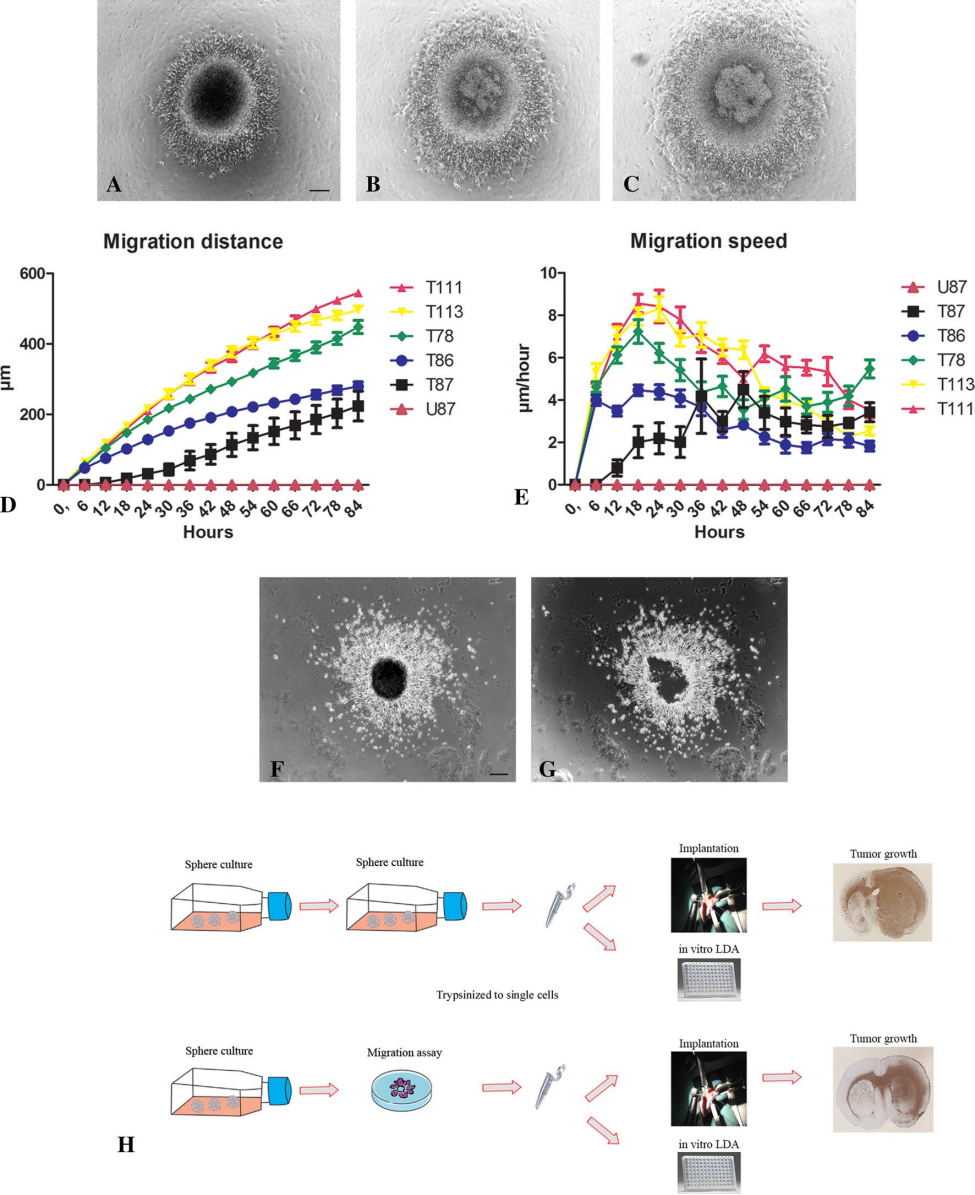
Migration assay based on stem cell medium and patient-derived spheroid cultures. Spheroids from the T78 culture were placed in Geltrex coated wells and a pronounced migration was observed after 24 h (**a**), 48 h (**b**) and 72 h (**c**). The migration distance (**d**) and speed (**e**) illustrated for five different spheroid cultures (T78, T86, T87, T111 and T113) and the commercial cell line U87MG, which was followed for 5 days with time-lapse microscopy. The migration assay allowed easy isolation of migrating cells for further studies. In cultures where spheroids had started to migrate (**f**), the spheroid was removed leaving the migrating cells behind (**g**) as illustrated for the T111 culture. Free floating spheroids and migrating cells were used for in vitro and in vivo limiting dilution assays (**h**). *Scale bar* 50 µm

The migration assay was a flat surface migration assay, which allowed migration to be monitored and migrating cells to be isolated for further investigations. Geltrex (Gibco) and serum-free medium was mixed (1 + 49) and 1.4 ml was added to each well in 12-well plates. Coated plates were incubated over night at 36 °C and the following morning the supernatant was aspirated. One spheroid (100–200 µm) was aspirated into a 0.1–2.0 µl pipette and placed on the coating. After incubating the plate for 75 min at 36 °C, 1000 µl serum-free medium was added. Each spheroid was monitored with time lapse microscopy over 5 days. We hypothesized that at the highest migration speed, genes had reached the highest level of deregulation. Migration speed was therefore calculated to find the optimal time point to isolate the migrating cells. At high migration speed, the migrating cells were isolated by removing the “central” non-migrating cells/spheroid with a micro-pipette (Fig. 1). The migrating cells were then washed twice in phosphate buffered saline before they were frozen as a pellet until further gene analysis. Migrating cells were trypsinized to single cells prior to use in other assays both in vitro and in vivo.

A set of GBM spheres from all five patient-derived cultures were fixed with 4 % formalin and paraffin embedded before immunostaining for CD133 and Sox-2. The corresponding migrating cells were trypsinized to single cells and re-cultured in neural stem cell medium. The formed spheres were fixed and paraffin embedded for immunostaining.

### Immunohistochemistry

Immunostaining of paraffin embedded spheroids were performed on 3 µm paraffin sections. Sections were deparaffinized and stained with CD133 (Miltenyi Biotec, clone W6B3C1; 1 + 40), and Sox-2 (R&D Systems, clone 245610; 1 + 400). The poly envision system was used for detection.

Mouse brains were before paraffin embedding manually cut in 1 mm coronal sections, which were cut in 3 µm paraffin sections and immunohistochemically stained with a Vimentin antibody (Nordic Biosite, clone EP20; 1 + 200). The poly envision system was used for detection.

### Automated quantitative analysis

Immunohistochemically stained slides were scanned on a Hamamatsu whole-slide scanner using NanoZoomer 2.0HT software (Hamamatsu, Ballerup, Denmark). The digital images were imported to the Visiopharm software module (Visiopharm, Hørsholm, Denmark). A computer-based software classifier within the Visiopharm software module was trained to identify specific staining and avoid background staining for each of the chromogenic stainings. The computer-based classifier calculated the area fraction of tumor cells expressing the stem cell marker of interest (CD133 and Sox-2).

### In vitro limiting dilution assay

Both free floating spheroids and the corresponding migrating cells from all five different patient-derived GBM spheroid cultures (T78, T86, T87, T111 and T113) were used for in vitro limiting dilution assays (LDA) performed as previously described [20, 21]. Spheroids and migrating cells were trypsinized to single cells and seeded in decreasing plating density using 96 well plates. After 10 days the percentage of wells not containing spheroids for each cell plating density was calculated and plotted against the numbers of cells per well. Data was interpreted in ELDA: Extreme Limiting Dilution Analysis software [22]. All experiments were performed in duplicate.

### Xenograft model

The use of mice in the present study was approved by The Animal Experiment Inspectorate in Denmark (permission J. Nr. 2013/15-2934-00973). Female Balb c nu/nu mice 7–8 weeks of age were anesthetized by a subcutaneous injection with a mixture of Hypnorm and Dormicum (0.12 ml/10 g). The mice were placed in a stereotactic frame on a heating pad. A midline incision exposing bregma was made. A burr hole 1 mm anterior and 2 mm lateral to bregma was made. A syringe with a blunt needle was inserted 3 mm into the brain. Cells were injected slowly into the brain over several minutes, while the needle was slowly removed to prevent a vacuum causing the tumor cells to escape. The skin was sutured with resorbable sutures.

The in vivo limiting dilution assay was performed using the patient-derived GBM spheroid culture T87. The intra-cerebral growth pattern and growth rate of this culture were known from a previous study in Balb c nu/nu mice [23]. Mice were injected with tenfold decreasing concentrations of free floating sphere cells (300.000 (n = 7), 30.000 (n = 7), 3.000 (n = 7)) and migrating cells (300.000 (n = 7), 30.000 (n = 7), 3.000 (n = 7)). Two mice died from anesthesia in the 30.000 sphere group.

Mouse health status was monitored daily and weight was measured twice per week. If any signs of neurological deficit were observed or weight loss more than 20 %, the mice were euthanized in a carbon dioxide chamber. When a single mouse showed symptoms, the whole group was euthanized. To evaluate early tumor size in all groups we chose to euthanize two mice in all groups when the first group showed symptoms. The brains were immediately removed and fixated in 4 % formalin for 48 h.

To assess tumor volume all slides were scanned with the NanoZoomer 2.0-HT slide scanner, Hamamatsu. Applying Simpson’s rule [24] the tumor volume was calculated by summing the tumor areas from all 1 mm coronal slices. This area was measured using the freehand area tool in the NanoZoomer Digital Pathology V2.3.11 (Hamamatsu).

### Statistics

Data was analyzed in GraphPad Prism version 5.01. The comparison of area fraction in the core and periphery was performed using an unpaired *t* test. Comparisons of in vivo results were performed using 1-way Anova and unpaired *t* test. Statistical significance was defined as p < 0.05.

### Gene expression profiling of cell lines

RNA from different GBM free floating spheroid cultures and corresponding migrating cells were purified using RNeasy system (Qiagen) and analyzed for global gene expression using Affymetrix 133 Plus 2.0 microarrays according to manufacturer’s guidelines. Raw CEL file data was normalized using the quantile normalization method in the free R package *limma* [25] using all genes in the data set. The normalized data was used for calculating the paired fold change (FC) between migrating cells and spheroids, considering genes with FC ≥2 and FC ≤0.5 as being up- or downregulated in the migrating cell lines, respectively.

The normalized data was log2 transformed, and differential gene expression analysis was performed using a paired *t* test. With the log2 transformed gene expression data, each spheroid culture and migrating cell sample was classified according to the four molecular GBM subtypes based on maximal positive correlation to the respective subtype centroids previously established by Verhaak et al. [19].

The gene expression pattern of the 840-gene signature established by Verhaak [19] was visualized using the heatmap2 function in the *gplots* R-package. The genes were grouped according to subtype cluster where genes constituting the Neural, Classical, Proneural and Mesenchymal subtype were colored green, blue, red and yellow, respectively. Spheroid cultures and migrating tumor cells classified as Neural, Classical, Proneural and Mesenchymal subtypes were colored green, blue, red and yellow, respectively.

## Results

All five GBM spheroid cultures showed pronounced migration (Fig. 1a–c). The highest migration distance was measured for T111, T113 and T78 (Fig. 1d). T87 had the lowest migration distance (Fig. 1d). Peak migration speed was found for T111, T113, T78 and T86 cultures after 18 h, which is in contrast to T87 peaking after 48 h (Fig. 1e). We also evaluated the migration of the commercial cell line U87MG but without identifying any migrating cells (Fig. 1d).

**Fig. 2:**
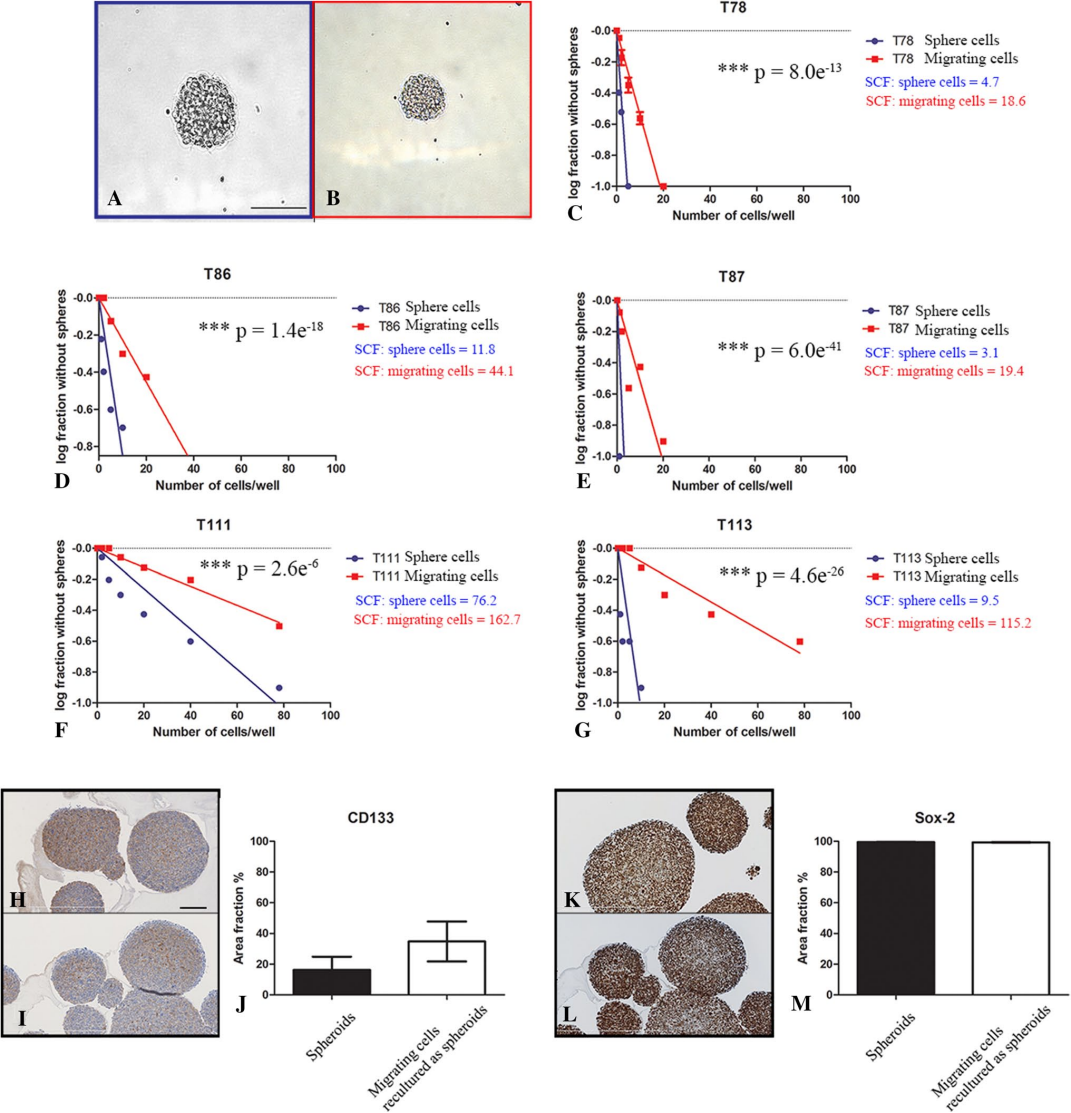
Comparison of spheroid formation and expression of stem cell markers for cells derived from free floating spheroids versus migrating cells. Spheroids were formed both upon trypsinization of spheres (**a**) and migrating cells (**b**) to single cells. The in vitro LDA revealed that both single cells derived from spheroids and migrating cells were capable of forming new spheroids (**c**–**g**), Statistical significance (p) was investigated with Extreme Limiting Dilution Assay (ELDA) software. Spheroids derived from an earlier passage of spheroids versus migrating cells were processed and histological sections stained with CD133 (**h, i**) and SOX-2 (**k, l**). Both stainings were quantified by software-based image analysis CD133 (**j**) and SOX-2 (**m**). Data are shown as means ± SEM, n = 5, comparisons were made with student’s *t* test. *SCF* Stem cell frequency. *Scale bar* 50 µm (**a, b**), 40 µm (**h, i, k, l**)

The in vitro limiting dilution assay (LDA) revealed that both spheroids and migrating cells trypsinized to single cells were capable of forming spheroids. Spheroids were formed at clonal cell concentration for GBM spheroid culture T78, T87 and T86 (both sphere and migrating cells) and T113 (sphere cells) but not for the T111 (Fig. 2a–g). For all five spheroid cultures, free floating spheroids had a significantly higher ability to form spheres compared to migrating cells. The T113 culture showed the largest difference of all cultures between free floating spheroids and migrating cells (Fig. 2g). We found a similar immunohistochemical expression of the stem cell markers CD133 and Sox-2 in spheroids derived from free-floating spheroids versus spheroids derived from migrating cells (Fig. 2h–m).

**Fig. 3:**
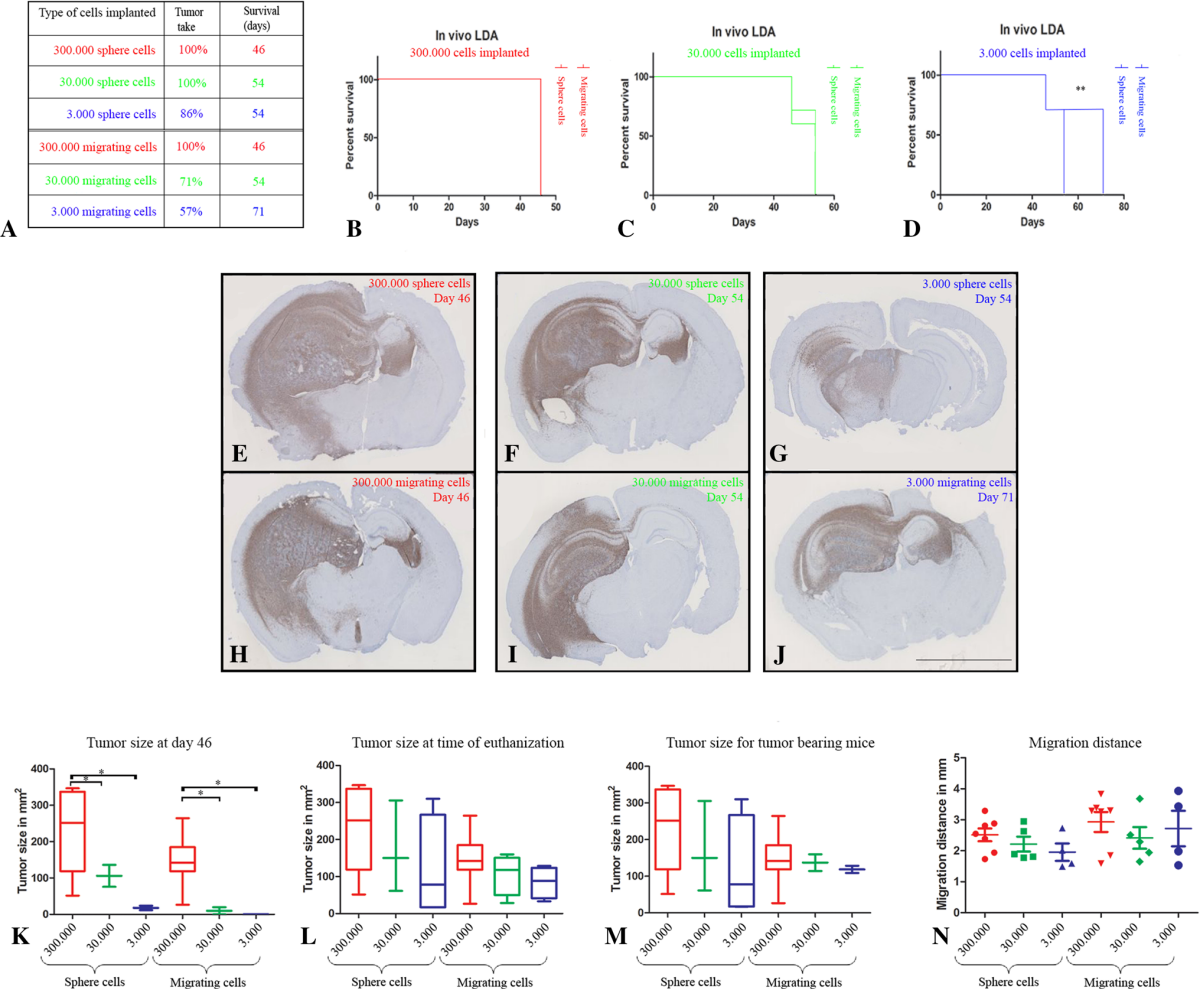
Comparison of in vivo tumor growth and survival for mice implanted with cells derived from free floating spheroids versus migrating cells. Six groups of mice were implanted with glioma cells from either spheroids or migrating cells at decreasing cell density (**a**). The survival is illustrated with Kaplan–Meier survival curves for orthotopically xenografted mice: 300.000 cells (**b**), 30.000 cells(**c**) and 3.000 cells (**d**). Only mice implanted with 3.000 migrating cells had a significantly longer survival than mice implanted with the corresponding number of sphere cells (**d**). Brains from the different groups were processed and histological sections stained with anti-human specific vimentin immunohistochemical staining for visualization of tumor size and migration pattern (**e**–**j**). Mean tumor size at day 46 for mice implanted with 300.000 (n = 7), 30.000 (n = 2) and 3.000 (n = 2) sphere and migrating cells (**k**). Mean tumor size at time of euthanization due to symptoms for mice implanted with 300.000 (n = 7), 30.000 (n = 3 for sphere group and n = 5 for migrating group) and 3.000 (n = 5) sphere and migrating cells (**l**). Mean tumor size for tumor bearing mice upon symptoms for mice implanted with 300.000 (n = 7), 30.000 (n = 3 for sphere group and n = 2 for migrating group) and 3.000 (n = 4 for sphere group and n = 2 for migrating group) sphere and migrating cells (**m**). Maximal migration distance for mice implanted with 300.000 (n = 7), 30.000 (n = 3 for sphere group and n = 4 for migrating group) and 3.000 (n = 4) sphere and migrating cells (**n**). Data were shown as means ± SEM, comparison was made with 1-way Anova and unpaired *t* test. *Scale bar* 5 mm (**e**–**j**)

In the in vivo LDA (Fig. 3a), mice injected with 300.000 sphere and migrating cells revealed symptoms at day 46. Therefore, these mice and two mice from the remaining groups were euthanized at that time point, where none of the other mice had neurological symptoms or weight loss. This was done to evaluate tumor size across all groups. Mice injected with 30.000 sphere and migrating cells and 3.000 sphere cells revealed symptoms at day 54, which was 1 week later than mice injected with 300.000 sphere and migrating cells. Mice injected with 3.000 migrating cells revealed symptoms at day 71 (Fig. 3b–d). Only mice injected with this number of migrating cells had a significantly longer survival compared to the corresponding sphere group, which revealed symptoms at day 54 (Fig. 3d).

Tumor size in mice implanted with 300.000 sphere cells after 46 days was significantly larger compared to mice implanted with 30.000 and 3.000 sphere cells (Fig. 3e, h, k). Same results were obtained for mice implanted with 300.000 migrating cells compared to mice implanted with 30.000 and 3.000 migrating cells (Fig. 3e, h, k). The tumor size in groups implanted with cells from spheroids was not significantly larger than groups implanted with migrating cells (Fig. 3k). Tumor size at time of euthanization of the individual groups tended to decrease with cell numbers injected (Fig. 3e–j, l). No significant difference in tumor size was observed comparing all tumors per group (Fig. 3m) or comparing the mean size of all tumors derived from spheroids versus tumors derived from migrating cells. The measured migration distance revealed that tumors derived from migrating cells had a longer, but not significant mean migration distance compared to the corresponding tumors derived from spheres (Fig. 3n).

Profiling of mRNA revealed in general similar levels of CSC-related mRNAs in spheres and their corresponding migrating cells (Table 1). Only Bmi-1 was significantly upregulated in migrating cells (1.2 fold). However, the upregulation was not significant when adjusting for multiple testing. ID1 revealed the highest fold change being 5.4 fold higher in migrating cells. In the HOX-gene-list HOXA3 was significantly upregulated in migrating cells (1.1 fold) (Table 1) but not when adjusting for multiple testing. In total the microarray platform revealed the expression of 23.160 genes. There was no significantly different mRNA expression between spheroids and migrating cells when adjusting for multiple testing. 

**Table 1:** List of 16 selected CSC markers and the HOX-gene CSC related list investigated at mRNA levels

CSC related genes	Fold change	P values	FDR	HOX gene list	Fold change	P values	FDR
EGFR	0.792	0.059	0.642	LOC400043	0.912	0.117	0.704
Nestin	0.898	0.062	0.645	HOXD8	0.922	0.147	0.720
CD36	0.950	0.474	0.882	HOXD10	0.940	0.460	0.879
Musashi-1	0.954	0.456	0.877	HOXA5	0.950	0.461	0.879
NANOG	0.966	0.617	0.923	HOXA2	0.950	0.455	0.877
ALDH1	0.988	0.589	0.916	TSHZ2	0.958	0.586	0.915
C-Met	0.994	0.716	0.945	HOXA7	0.968	0.400	0.859
Podoplanin	1.016	0.852	0.972	HOXD4	0.989	0.741	0.950
SOX2	1.017	0.370	0.844	HOXC6	1.005	0.985	0.997
Integrin α6	1.018	0.863	0.975	FAM110C	1.009	0.929	0.987
CD44	1.054	0.908	0.984	HOXA10	1.013	0.767	0.955
CD133	1.061	0.764	0.954	PROM1	1.061	0.764	0.954
OCT4	1.097	0.475	0.882	HOTAIR	1.076	0.313	0.820
CD15	1.130	0.118	0.704	LOC375295	1.089	0.972	0.995
BMI1	1.196	0.004	0.553	SKAP2	1.120	0.091	0.677
ID1	5.419	0.066	0.655	HOXA3	1.125	0.005	0.553
				HOXA10	1.186	0.067	0.656

Left data set represents 16 stem cell/CSC related genes for spheres and isolated migrating cells. Right data set represent the HOX-genes. The data revealed no significant change in mRNA expression in migrating cells compared to GBM spheres when adjusting for multiple testing. Listed according to fold change

**Fig. 4:**
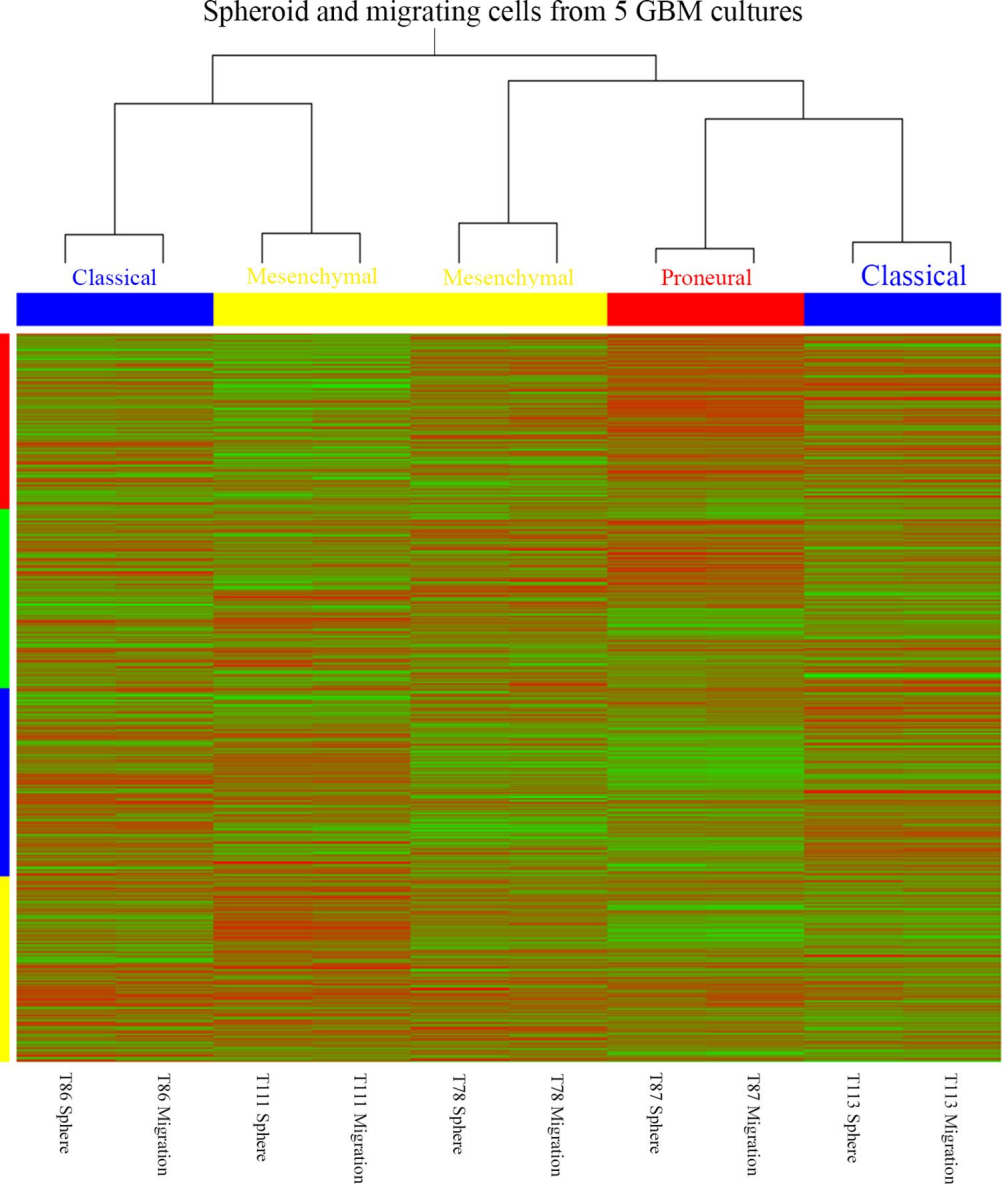
Heatmap of mRNA profiling results obtained with five different GBM spheroid cultures and corresponding migrating cells. Hierarchical cluster analysis revealed that GBM spheroid cultures and corresponding migrating cells all clustered with each other. Molecular subtyping revealed three different subtypes: Classical, Mesenchymal and Proneural. Migration did not induce a shift in subtype

The subtypes estimated were similar for each of the different patient-derived GBM spheroid cultures and their corresponding migrating cells (Fig. 4).

## Discussion

In line with the well-known migration for GBMs in the brain, pronounced migration was observed for all investigated GBM spheroid cultures. Supporting the in vivo-like features and translational potential of our model, migration was not observed for the standard U87MG cell line, thereby confirming the growth pattern for U87MG earlier found in the orthotopic xenograft model, where U87MG cells form a circumscribed tumor with a sharp border between tumor and parenchyma [17]. Our major novel finding in vitro was that not only the five GBM spheroid cultures but also the corresponding in vitro isolated migrating cells had the capacity to form spheroids. Furthermore, there was a similar expression of the stem cell markers CD133 and Sox-2 in spheroids derived from a previous passage of spheroids as well as migrating cells. The reduced but still preserved spheroid formation capacity of migrating cells compared to spheroid cells was significant for all five GBM spheroid cultures. Piccirillo et al. suggested the existence of only a very poor CSC population in the tumor periphery using surgical biopsy material from the periphery of GBMs. Cells obtained from the periphery in that study were unable to form spheroids in long-term culturing conditions compared to tumor cells isolated from the core, but orthotopic tumor formation in mice was observed [26]. This discrepancy might to some degree be explained by critical isolation and survival conditions for tumor cells in the surgical biopsy material as well as our migration assay over-preserving stem cell features of migrating cells leading to enhanced high spheroid formation and tumorigenic capacity of migrating cells. Supporting this, Wang et al. has earlier demonstrated that CD133^−^ can give rise to CD133^+^ tumor cells upon culturing in serum-free medium [27].

In the xenograft model the tumor take was lower in mice implanted with 30.000 and 3.000 migrating cells compared to mice implanted with 30.000 and 3.000 sphere cells. Correspondingly, the overall survival was significantly higher in mice implanted with 3.000 migrating cells compared to mice implanted with 3.000 sphere cells. This is in line with our in vitro LDA results. The migrating tumor cells thus preserved a tumorigenic potential, although at a lower level than the corresponding sphere cells. This was supported by Piccirillo et al. who demonstrated that tumor cells from the periphery of GBM patients could give rise to tumor formation upon xenografting, although the tumorigenic capacity was reduced [26]. The reduced tumorigenic capacity of migrating tumor cells might be explained by the lack of a stimulating microenvironment. Hypoxia is known to favor the CSC potential [18, 28, 29] and could explain that the highest tumorigenic potential was found for spheroids [18]. It might also be explained by comparison of the tumorigenic capacity of migrating tumor cells with the tumorigenic capacity of free floating spheroids instead of the residual spheroids left after migration. The residual spheroids were difficult to separate precisely from the migrating cells since migrating cells should be avoided in these samples. Residual spheroids were therefore not considered to be suitable for comparison.

The migrating cells preserved the spheroid level of CSC mRNAs both focusing on the 16 different CSC-related markers as well the HOX-gene list [30]. Bmi-1 and HOXA3 were found to be upregulated in the migrating cells, but not significantly when adjusting for multiple testing. Compared with the in vitro and in vivo LDA assays a slightly reduced mRNA level was expected at least for some CSC-related mRNAs. This was not found and may be explained by lack of sensitivity of the profiling platform. Additionally, the mechanisms that regulate migration may differ dependent on the molecular subtype of GBM. This may furthermore suggest that future investigations of mechanisms involved in migration should be investigated in several cultures of each molecular subtype to identify the responsible and targetable mechanisms. The mRNA profiling suggested that migration did not influence GBM molecular subtype. Our data suggested that the Mesenchymal subtype has the highest migration speed followed by the Classical subtype. Although this is an interesting finding, this should be further investigated in a larger study. None of “Verhaak’s” subtypes have to our knowledge previously been associated with a higher migration potential [19, 31]. Importantly, all subtypes have the potential to infiltrate the normal brain parenchyma [32].

## Conclusion and perspective

The obtained results suggest that migrating GBM cells preserve expression of stem cell markers as well as their tumorigenic capacity independent of GBM subtype, although the tumorigenic capacity of migrating tumor cells is slightly reduced. Glioma stem cells have been reported to be resistant to both chemotherapy and radiation [7] and since glioma cells in the periphery display a CSC profile, this might explain the limited response to radio- and chemotherapy and therefore be a major reason for treatment failure and a poor overall survival. The presence of CSCs in the periphery after optimal neurosurgical resection should therefore be taken into account in the future development of targeted therapies against both CSCs and non-CSCs. The established migration assay preserving CSC features of migrating glioma cells is of potential value for discovery of novel targets in this critical cell population.
